# The mind, brain, and body study: A protocol for examining the effects of the gut-brain-immune axis on internalizing symptoms in youth exposed to caregiving-related early adversity

**DOI:** 10.1016/j.bbih.2024.100880

**Published:** 2024-10-05

**Authors:** Shiba M. Esfand, Francesca R. Querdasi, Naomi N. Gancz, Paul W. Savoca, Siyan Nussbaum, Jennifer A. Somers, Julia Ditzer, Matthew B. Figueroa, Kristen Chu, Emily Towner, Bridget L. Callaghan

**Affiliations:** a Department of Psychology, University of California, Los Angeles, 502 Portola Plaza, Los Angeles, CA, 90095, USA; b Department of Psychology, Clinical Child and Adolescent Psychology, Technische Universität Dresden, Dresden, Germany; c Department of Psychology, University of Cambridge, Cambridge, United Kingdom; d Department of Psychological Sciences, Auburn University, 111 Thach Hall, Auburn, AL, 36849, USA

**Keywords:** Care-giving related adversity, Gut-brain-immune axis, Internalizing symptoms, Memory and reward systems

## Abstract

Experiences of caregiving-related adversity are common and one of the strongest predictors of internalizing psychopathology (i.e., anxiety and depression). Specifically, individuals who have been exposed to such early adversities have altered affective neurodevelopment, impaired memory systems, increased risk of developing internalizing disorders, greater inflammation, and differences in gastrointestinal (gut) microbiome composition. Crucially, the gut microbiome undergoes a sensitive period of development that precedes neural and immune sensitive periods, thus making it a potentially fruitful target for intervention. Though previous work has assessed neural, immune, and gut microbiome systems in individuals exposed to early adversity, studies have primarily looked at these biological systems independently. The Mind, Brain, and Body study (MBB) implements multimodal and longitudinal design to assess how changes in the gut microbiome following caregiving-related adversity may underlie altered affective neurodevelopment, memory, and immune functioning in youth and contribute to internalizing symptoms. Across three waves, spread approximately 12–18 months apart, youth with and without previous experiences of caregiving-related adversity completed self-report measures of mental and physical health, provided stool, saliva, hair, and blood samples, and completed an MRI scan. Results of this study will expand our knowledge on how the gut microbiome shapes several biological and cognitive systems and motivate future work investigating the gut microbiome as potential target for intervention.

## Highlights

• Early adversity (EA) increases risk for developing internalizing disorders.
• Early adversity has also been linked to changes in the gut-brain axis.
• The Mind, Brain, Body study tests gut-brain links to internalizing disorders after EA.
• Results will inform an understanding of gut-brain axis involvement in mental health.

Exposure to early life adversity (ELA) is one of the strongest predictors of psychopathology in youth and across the lifespan (Kessler et al., 2010). Such ELAs vary in their form and source, which may have unique impacts on development (McLaughlin and Sheridan, 2016). ELAs that involve the caregiver have particularly potent impacts on well-being, including emotional, psychological, and physiological system function (Callaghan et al., 2020; Gee, 2021; Slopen et al., 2012). Such caregiving-related early adversities (crEA) involve interruptions, separations and/or dysfunctions within the parent-child relationship, such as caregiver maltreatment, as well as institutionalized or foster care (Tottenham, 2020). These crEAs increase risk for mental illness in youth, potentially via their impacts on the amygdala and hippocampus (Vannucci et al., 2023; VanTieghem et al., 2021). One underexplored possibility is that crEAs may increase the risk for youth psychopathology through a widespread impact on both central (i.e., brain) and peripheral (e.g., microbiome and immune) systems that are involved in health. Emerging research (Acheson et al., 2012; Zlomuzica et al., 2014) highlights the importance of central and peripheral system interactions in an individual's health, for instance, through the brain-gut-microbiome axis. Thus, research on the wide-reaching impact of crEAs across the brain and body may reveal novel mechanistic pathways through which health risks operate, and further, targets for personalized interventions and preventions. We designed the Mind, Brain, and Body study to assess the impact of crEAs on the brain-gut-microbiome and immune axis across childhood and through adolescence.

## 1 Impact of crEAs on cognitive and emotional development

Childhood and adolescence are developmental periods defined by heightened cognitive and emotional plasticity (Kadosh et al., 2013). As such, it is perhaps not surprising to see that exposure to ELA is linked to widespread alterations in cognitive and emotional development. Children exposed to crEAs have been shown to display deficits in inhibitory control, theory of mind, forming memories of socioemotional stimuli, and understanding emotions of other children (Rifkin-Graboi et al., 2021; Tarullo et al., 2007). CrEA exposure is also associated with deficits in reward processing, including hyposensitivity to rewards, reward responsivity, and reward learning (Guassi Moreira et al., 2022; Oltean et al., 2023; Sheridan et al., 2018). Critically, these cognitive and emotional outcomes after crEA exposure may continue to be malleable across the adolescent period of development with continuing environmental input. For instance, the effects of crEA exposure on reward processing were ameliorated by experiences of higher quality caregiving in adolescence (Colich et al., 2021). Together these results demonstrate the pervasive effect of crEA exposure on cognitive and emotional development, and the importance of investigating factors that can potentially moderate the outcomes of crEA exposure across development.

As internalizing psychopathology (e.g., anxiety and depression) is especially high after crEA exposure (Buthmann et al., 2022; Gee, 2021; Green et al., 2010), it is important to consider how crEA affects the development of cognitive and emotional features which are core to the phenomenology of internalizing disorders. One core phenotype of internalizing disorders is memory dysfunction, namely persistent and intrusive memories of potential threat (Acheson et al., 2012; Zlomuzica et al., 2014). Memory dysfunction has been shown to be impacted by crEAs. For example, rodents who experienced maternal separation (a model of crEA) displayed greater retention of fear-related memories compared to those who had standard rearing (Callaghan and Richardson, 2012; Cowan et al., 2016). Among human children, exposure to crEAs has been found to impact affective memory formation. For instance, in a study assessing false recognition of events, Vannucci and colleagues found that children exposed to crEAs (vs non-exposed) were more likely to falsely endorse watching a scene that involved a negative parent-child interaction (Vannucci et al., 2023). Among youth who experienced institutional rearing, reductions in executive functioning, specifically working memory, have been shown to mediate the relationship between previous institutionalization and development of ADHD symptoms (Tibu et al., 2016). While these studies suggest memory as one mechanism that might underlie increased internalizing psychopathology after crEA exposure, most studies investigating crEA impacts on memory have been cross sectional and have not often linked memory to internalizing psychopathology. The current study was designed to fill in those gaps by longitudinally investigating both memory function and internalizing symptoms in adversity exposed youth across childhood and into adolescence.

Reward functioning is another critical domain of cognition and affect that has been associated with internalizing disorders and impacted by crEA exposure. For instance, crEA exposed children have been shown to exhibit reduced reward learning compared to non-exposed children (Hanson et al., 2017; Weller and Fisher, 2013; Wilkinson et al., 2021), and reduced motivation to obtain rewards (DelDonno et al., 2019; Guyer et al., 2006; Kautz et al., 2020). Moreover, crEAs have been associated with greater impulsivity and risk taking (Herrenkohl et al., 2010; Kamkar et al., 2017; Oshri et al., 2018). Similar to the memory literature, however, few studies examining crEA impacts on reward function have looked at outcomes longitudinally, nor made links to internalizing symptoms. The current study has attempted to fill in those gaps by assessing reward learning, and reward related neural function, in adversity exposed youth across childhood and into adolescence.

As memory and reward systems are heavily implicated in the development and progression of internalizing disorders (Aikins and Craske, 2001; Craske et al., 2011; Dillon et al., 2014), elucidating how memory and reward systems are altered in the context of crEA, especially during high-risk times for emergence of internalizing psychopathology (middle childhood through adolescence) may help to identify specific mechanisms of intervention.

## 2 Impact of crEAs on neurodevelopment

The impact of crEAs on brain development appear to be particularly prominent in neural regions important for the processing of affective information, e.g., the amygdala, hippocampus, prefrontal cortex, and striatum (VanTieghem et al., 2021). It has been suggested that such associations exist because these brain regions are undergoing rapid maturation and are highly plastic in early development (Bayassi-Jakowicka et al., 2021), when they may be more sensitive to caregiver cues (Tottenham, 2020). Indeed, parents have been shown to be powerful regulators of their children's physiological and neural development, with studies suggesting that childhood may be a sensitive developmental period for parental regulation over neural function (Gee et al., 2014; Nachmias et al., 1996; Seltzer et al., 2012).

In terms of the impact of crEAs on affective circuit development, both rodent and human studies have found alterations in dopaminergic pathways, such as reduced activation in the ventral striatum following adversity exposure (Dillon et al., 2009; Fulford and Marsden, 1998; G. H. Jones et al., 1992). CrEAs have also been associated with reduced amygdala, medial prefrontal cortex (mPFC), and hippocampal volume (Dannlowski et al., 2012; McLaughlin et al., 2019; van Velzen et al., 2016; VanTieghem et al., 2021), and altered connectivity between the amygdala and the medial prefrontal cortex (mPFC) in humans (Marusak et al., 2016). One systematic review (N = 109 studies) found that children exposed to crEAs display altered amygdala-PFC connectivity (McLaughlin et al., 2019). Similar results were found in a separate meta-analysis of 46 studies, such that reduced amygdala-PFC connectivity was found among children and adults who were exposed to crEAs (compared to non-exposed participants; (Marusak et al., 2016). This decreased amygdala-mPFC connectivity has been shown to impact memory formation (Feng et al., 2014). Relatedly, children exposed to crEAs display reduced hippocampus-PFC connectivity compared to controls (McLaughlin et al., 2019). Moreover, crEA exposure has been extensively associated with alterations in amygdala and hippocampal plasticity, namely accelerated maturation of both regions (Gee et al., 2013; Herzberg et al., 2021; Silvers et al., 2016). This accelerated maturation of affective circuitry has been theorized to slow the maturation of other neural networks (Herzberg et al., 2021). Thus, further elucidation of crEAs downstream effects on affective networks, and other neural biological systems is needed.

One reason that affective circuitry may be especially affected by crEA exposure is that many of the nodes in these circuits harbor a high number of stress hormone receptors and are involved in secretion of stress-related hormones through their involvement in the hypothalamic pituitary adrenal (HPA) axis. Both animal and human models have implicated the amygdala and hippocampus in the secretion of stress-related hormones, such as cortisol (Hakamata et al., 2017; Pagliaccio et al., 2014; Vaisvaser et al., 2013). Interestingly, VanTieghem and colleagues found that reduced amygdala and hippocampal volume was predictive of greater cortisol levels in crEA exposed youth (VanTieghem et al., 2021).

Though extensive evidence suggests that crEAs are associated with altered development and functioning of the central nervous system (CNS), less is known on how the CNS interacts with peripheral biological systems, such as the HPA axis, the gut microbiome, or the immune system, to impact the development of affective psychopathology, particularly after experiences of crEA.

## 3 Impact of crEAs on peripheral changes in immune-related biomarkers and the gut microbiome

While perhaps the majority of research on biological outcomes associated with crEA exposure have focused on the central nervous system, crEAs and ELA more broadly are also known to have an impact on peripheral biological processes, including the immune system and the microbiome. For instance, in a sample of young adults it was shown that adversity exposure interacted with adiposity to predict IL-6 reactivity (a pro-inflammatory cytokine), such that ELA exposed adolescents with high adiposity had higher IL-6 reactivity than low ELA exposed adolescents (regardless of adiposity) and low adiposity ELA exposed individuals. A recent meta-analysis of nearly two hundred studies, found that experiencing ELA is consistently correlated with higher levels of inflammatory markers, and that the relationship between ELA and presence of inflammatory markers increase in magnitude across the lifespan (Chiang et al., 2022). Among adults, those who were separated from their biological parents and were later adopted, relative to those who were reared by their biological parents, had a greater risk of experiencing T-cell immunosenescence, a condition wherein T-cells are less effective in identifying invading agents (Elwenspoek et al., 2017), and increased susceptibility to autoimmune agents (Rodriguez et al., 2021). Indeed, hospitalization due to autoimmune diseases in adulthood is linked to childhood experiences of ELA (Dube et al., 2009; Macarenco et al., 2022). Critically, immune functioning has also been linked to mental health outcomes (Nusslock and Miller, 2016). A recent meta-analysis of 28 studies found a significant positive association between elevated peripheral cytokine levels and presence of pediatric internalizing disorders (Howe and Lynch, 2022). Though these aforementioned studies have linked crEAs and immune functioning, much remains to be understood about how crEAs influence immune processes across development and contribute to the emergence of internalizing symptoms.

Related to the immune system, the gastrointestinal (i.e. gut) microbiome is another biological system known to be altered by the early life environment. Critically, the gut microbiome may be the missing link in elucidating pathways by which exposure to adversity alters neural, immune, and psychological functioning. The gut microbiome, or the trillions of microorganisms that live in our gut, is responsible for regulating several aspects of health including defense against pathogens and metabolism of key nutrients (Cho and Blaser, 2012). Moreover, as the intestine is the largest immune interface in the human body (Takiishi et al., 2017), the interactions between the immune system and microbes are extensive (Zheng et al., 2020), highlighting the importance of considering both systems in understanding peripheral influences over mental health. Indeed, several studies in rodents and humans have shown that the gut microbiome plays a causal role in affective symptoms (Hao et al., 2020; Kelly et al., 2016; Peirce and Alviña, 2019; Ritz et al., 2024), and have highlighted different microbiome profiles associated with internalizing symptoms and disorders (Barandouzi et al., 2020; Butler et al., 2023; Kouraki et al., 2023). Relatedly, the oral microbiome has also been found to impact internalizing symptoms and inflammatory pathways (Martínez et al., 2021; Simpson et al., 2020; Wingfield et al., 2021), perhaps through interactions with the gut microbiome via a wider brain-oral-gut-microbiome axis (Bowland and Weyrich, 2022). Thus, the oral microbiome community may serve as another crucial biological system that contributes to the onset of internalizing disorders. While the evidence for a link between the microbiome and mental health is now quite strong, the majority of studies have focused on adulthood, despite the developmental nature of anxiety and depression (Kessler et al., 2010), and despite the fact that the microbiome exhibits dramatic developmental change into adolescence (Burcham et al., 2020; Korpela et al., 2021). Moreover, most microbiome studies have concentrated on characterizing its taxonomy (achieved through 16s rRNA sequencing), rather than its functional potential (i.e., an estimate of all metabolic functions in a sample that can be assessed via whole genome sequencing) or the influence of microbial products or outputs (e.g., through measuring microbially derived metabolites). As such, specificity in our understanding of how the microbiome contributes to the development of mental health is lacking, especially across development.

Notwithstanding the methodological limitations in the human literature, several studies have now linked experiences of ELA with altered gut microbiome composition across the lifespan. In one study, Hanstoo and colleagues found that pregnant women retrospectively reporting a greater number of ELA experiences, had differentially abundant taxa (namely *Prevotella*) in their guts, relative to pregnant women with lower ELA exposure. In that study, specific bacteria (i.e., *Bacteroides, Prevotella, Megasphaera)* were associated with key markers of immune functioning and stress (i.e., cortisol levels, IL-6, TNF-a) independent of ELA exposure. Earlier in development, the gut microbiome has been shown to be altered specifically after crEA exposure. For instance, in a small proof-of-concept sample, crEA exposure (previous institutionalization followed by international adoption) was associated with lower alpha diversity (i.e., a within-subjects measure of microbiota richness, ‘number of bacterial groups’) relative to a group of non-crEA exposed individuals, and crEA exposure explained significant variance in beta diversity metrics (Callaghan et al., 2020). Moreover, in that study, a number of differentially abundant microbes were associated with crEA exposure, including some (an unknown genus from the *Lachnospiraceae* family lower in the crEA group) that were associated with neural reactivity to stereotypical fear faces (positive associations with the lateral and medial prefrontal cortex, and precuneus/cerebellum, and negative associations with the post central gyrus). In another proof-of-concept study on a similar population of crEA exposed adolescents, no differences in alpha diversity between crEA and non-crEA exposed groups were reported, though beta diversity (a between-subjects measure of differences in microbiome composition) was high between the groups (Reid et al., 2021). Moreover, several microbes were differentially abundant between the two groups: *Prevotella, Bacteroides, Coprococcus, Streptococcus,* and *Escherichia* were all higher in crEA exposed youth. In some very recent studies, crEA has also been linked to reduced diversity, different community composition, more pathogenic taxa abundance, and less tonic cortisol responsive microbes, in the *oral* microbiome (Gancz et al., 2024), which has itself been linked to mental health in youth (as mentioned above; Simpson et al., 2020). As such, while the literature supports an impact of ELA on the composition of the microbiome in development, significant limitations lie in the small samples within the gut microbiota studies, and the lack of longitudinal data to examine within-individual change in various microbiome communities across developmental time. To address these limitations, our study will be the first to implement a longitudinal design to assess the prospective impact of crEA exposure on the gut and oral microbiome communities.

Intriguingly, several studies have demonstrated that a therapeutic focus on the gut-brain axis may be beneficial in addressing increased mental health risks posed by early adversity exposure. For instance, in rodent models, administration of probiotics to mothers and/or their offspring can reverse the impact of maternal separation (a rodent model of crEA exposure) on memory development (Cowan et al., 2016), even across generations (Callaghan et al., 2016), depression and anxiety behavior (Dandekar et al., 2022; De Santa et al., 2024; Peng et al., 2019; Zhu et al., 2022), as well as on pubertal timing (Cowan and Richardson, 2019), HPA axis reactivity (Fukui et al., 2018; Gareau et al., 2007; Peng et al., 2019), immune responses (Barouei et al., 2015; Desbonnet et al., 2010), gastrointestinal problems (Barouei et al., 2015; Gareau et al., 2007; Moussavi et al., 2023; Zhu et al., 2022), and on neural circuits underpinning fear expression and extinction (Feng et al., 2014). Given exposure to crEAs significantly increase the risk of developing, and the early onset of, internalizing disorders (Phillips et al., 2005; Scott et al., 2008), there is a pressing need to find effective treatments for internalizing disorders in youths who have experienced crEA. Critically, the efficacy rates of current evidence-based treatments for anxiety and depression in youth are poor (40–60%). As such, identifying mechanisms for internalizing disorders in youth, with the goal of translating findings to increase treatment efficacy, is a public health priority. In the Mind, Brain, and Body study those aims are addressed by examining the relationships between early crEA exposure, microbial, immune, neural, affective, and cognitive development, in a longitudinal sample of youth spanning middle childhood through adolescence.

## 4 The mind, brain, and body (MBB) study

The primary goal of the Mind, Brain, and Body study (MBB) was to identify links between early childhood crEA exposure with the development of gut microbiome functional potential and species-level composition, across middle childhood to adolescence, a time when the gut microbiome appears to still be developing (Agans et al., 2011; Flannery et al., 2019), but when there is a lack of detailed information on such development. In examining gut microbiome functional potential at three time points across middle childhood to adolescence, the MBB study goes beyond prior investigations which have focused on a taxonomic study of microbiome development cross-sectionally in both typically developing and adversity exposed samples (Callaghan et al., 2020; Korpela et al., 2021; Reid et al., 2021). As such these data will contribute to our understanding of microbiome maturation in the general population within the U.S. as well as in crEA exposed subgroups.

As a secondary goal, the MBB study aimed to provide an integrated understanding of how crEA-linked gut microbiome changes were associated with cognitive, affective, and neural development across middle childhood to adolescence. In particular, we were interested in the links between the gut microbiome and two different neural circuits: (1) the hippocampal based memory system and (2) the striatal based reward system. Interest in the hippocampal based memory system stemmed from the growing number of studies linking the microbiome with memory and hippocampal function that had been reported in bumblebees (Li et al., 2021), mice (D'Amato et al., 2020; Leigh et al., 2020; Olson et al., 2021), and within adult human populations (Kolobaric et al., 2024; Meyer et al., 2022; Oyarzun et al., 2022), including in the context of Alzheimer's Disease (Sochocka et al., 2019; Vogt et al., 2017). Critically, despite those associations, there is very little information on how the gut microbiome was linked to memory *development* across middle childhood and into adolescence. Although it is clear that the hippocampus continues to develop across those years (Callaghan et al., 2021), and is affected by adversity exposure (Lambert et al., 2019). Also, as memory function and the hippocampus are affected in a range of mental illnesses, we considered these as important systems to understand in the context of crEA associated mental illness risks, especially internalizing psychopathology (e.g., anxiety and depression).

In terms of the striatal based reward system, the gut microbiome has been linked to reward learning, the striatum, and reward-relevant dopaminergic neural systems in rats (Jadhav et al., 2018) and mice (Dohnalová et al., 2022; Shan et al., 2021), adult humans (García-Cabrerizo et al., 2021; Gupta et al., 2020; Jakobi et al., 2024), and in youth with ADHD (Aarts et al., 2017). However, similar to the hippocampus, little data exists on the gut microbiome links with striatal and reward *development* across middle childhood to adolescence, particularly in the context of adversity. This is so, despite dopaminergic striatal reward circuits showing pronounced development over adolescence, and being linked to mental health outcomes. Thus, together, the MBB study will contribute to a greater understanding of hippocampal based memory development and striatal based reward development in the middle childhood to adolescent years, how such development is affected by crEA exposure and linked to internalizing symptoms, as well as an understanding of how those features are associated with the gut microbiome. Additional analyses exploring the relationship between crEAs, oral microbiome composition, and neurodevelopment will also be conducted.

A final goal of the MBB study was to consider a range of variables that may moderate associations between adversity exposure and the other variables of interest in this study (microbiome, cognitive, affective, and neural development). Prime amongst those were inflammation, tonic cortisol levels (measured through hair), and normative variability in the parenting environment.

To achieve the stated goals, the MBB study involved three timepoints (waves) of data collection. Each wave was spaced 12–18 months apart in an accelerated longitudinal design. We recruited individuals aged 6–16 years at Wave 1, so that ages 6–18 years were included by the conclusion of Wave 3. The MBB study was funded by the National Institutes of Menta l Health (R00MH113821 to B.C), with additional support provided by a UCLA Faculty Career Development Award to B.C. and a UCLA Goodman-Luskin Microbiome Center Seed Fellowship Award to F.R.Q. and N.N.G. We indexed microbiome composition through shotgun metagenomic sequencing of stool samples, memory through performance on an autobiographical spatial associative memory task, internalizing symptoms through questionnaire assessments, and neural function through a spatial memory task with a reward component. In addition, we used indexed inflammation through dried blood spots, tonic cortisol through hair samples, and examined the parent-child relationship through behaviorally coding parent-child interactions while they were discussing a conflict and then a pleasant event.

### 4.1 Study aims & hypotheses

In Aim 1, we planned to test the stability of the composition of the gut-microbiome across the three study timepoints (Waves 1, 2, & 3) of data collection, between the two groups (crEA & Comparison). Based on earlier findings in our proof-of-concept sample (Callaghan et al., 2020), we hypothesized (H1) that we would observe lower taxonomic diversity in the crEA group relative to the comparison group, and less increase in that diversity over time. We also hypothesized that the crEA group would cluster separately from the Comparison group in terms of taxonomically defined beta diversity. At the time of study conception, we did not have specific hypotheses about the influence of crEA on the functional composition of the microbiome, or its change over time.

In Aim 2, we planned to test the association of crEA with internalizing symptoms and memory performance over time. Based on prior literature, we hypothesized (H2) that crEA would be associated with a greater expression of internalizing symptoms than the Comparison group at all time points (Callaghan et al., 2019), and differential performance on the autobiographical spatial memory task (Callaghan and Richardson, 2012; Lambert et al., 2019) over the study waves.

In Aim 3, we planned to test the association between the gut-microbiome at Wave 1 and developmental change in memory performance from Wave 1–3. We hypothesized (H3) that features of the gut-microbiome at Wave 1, especially those that were linked to inflammatory function, would predict change in memory performance from Wave 1 to Wave 3. We also hypothesized that the association between the microbiome/inflammation and memory performance would be mediated by hippocampal activity during learning in Wave 2. However, due to complications related to COVID-19, we did not collect MRI data in Wave 2 as planned, such that the final mediation hypothesis was not testable in this dataset. Though, we did collect neuroimaging data in Wave 3 on a spatial memory task (Parr et al., 2021) that had a reward component to further index the role that reward plays in these processes with crEA exposed youth.

Given the limited data directly linking the gut microbiome to striatal development and reward learning, we do not have specific hypotheses about the influence of crEAs on the gut-brain-reward axis. Thus we will conduct exploratory analyses on how crEA exposure impacts gut microbiome composition and how this may be related to striatal connectivity in the presence of rewards and levels of neuromelanin (a proxy measure of dopamine) in the locus coeruleus a key region associated with attention and stress responses that interacts with the hippocampus and amygdala (Tanaka et al., 2000).

### 4.2 Methods

All procedures were carried out in accordance with the University of California Los Angeles (UCLA) Institutional Review Board Committee who approved the study protocol.

#### 4.2.1 Participants

The target sample for enrollment in Wave 1 of this study was children (aged 6–9 years) and teens (aged 13–16 years) who had experienced some caregiving-related early adversity (e.g., maltreatment, extended parent separation), and a comparison group of children and teens who had not experienced those caregiving adversities (e.g., had always been with their birth/first/biological parents). Participants for both groups were recruited from flyers posted in the community through internet support groups and message boards for adoptive and foster families, advertising with community partners such as organizations that support foster and adoptive families, and online advertising (i.e., Meta-targeted advertisements). The target sample size for Wave 1 of this study was N = 150 youth, n = 75 in each of the crEA and comparison groups, evenly distributed across the two age bins (n = 37–38 in each of the child and teen groups for each of the caregiving classifications). The obtained sample size for Wave 1 was N = 163 from 119 families, which was over the target to account for missing questionnaire data. We also expanded our age inclusion criteria to capture 10–12 years of age due to the challenges of recruiting youth with crEA exposure. See Table 1 for the age, sex, and other demographic distribution for the sample in Wave 1. Full inclusion/exclusion criteria can be found in the *Supplement.*

**Table 1:** Sample Characteristics.

	Full Sample (N = 163)	Comparison (N = 90)	crEA-exposed (N = 73)	Test statistic	p-value
Early caregiving experiences					
Continuously raised by biological/birth parents - %	55.2	100.0	–		
Adopted at birth or placed in US foster care before adoption - %	25.2	–	56.2		
Experienced institutional/foster care outside of the US before adoption - %	8.6	–	19.2		
Placed in US foster care before entering a guardianship care arrangement - %	10.4	–	23.3		
Parental maltreatment not resulting in foster care - %	0.6	–	1.4		
Exposure to a potentially traumatic event - % Any exposure	26.4	3.3	54.8	69.79	<0.001

Demographics	Full Sample (N = 163)	Comparison (N = 90)	crEA-exposed (N = 73)	Test statistic	p-value
Child Age - M(SD)	11.18 (3.42)	11.20 (3.65)	11.15 (3.13)	0.087	0.465
Child Pubertal Status - % Pre-pubertal	36.2	42.2	28.8	0.869	0.351
Child Sex – % Male	50.9	54.4	46.6	0.999	0.318
Child Ethnicity – % non-Latinx	49.7	48.9	50.7	0.456	0.500
Child Race- % White	43.0	41.1	45.2	3.279	0.070
Primary Caregiver Ethnicity – % non-Latinx	71.2	63.3	80.8	9.95	0.002
Primary Caregiver Race - % White	57.7	42.2	76.7	27.996	<0.001
Primary Caregiver Education - % College Education or Above	65.0	68.9	60.2	0.090	0.764

*Note.* Demographics from youth and caregivers in Wave 1.

**Fig. 1:**
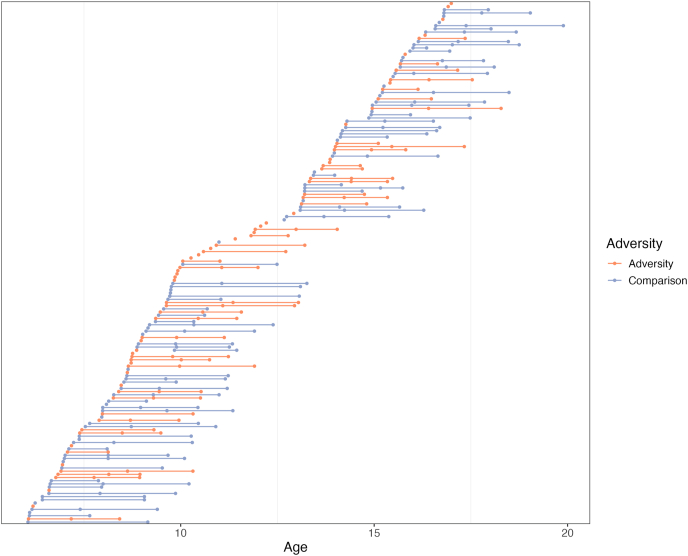
Attrition of participants across study waves (1–3), with blue representing the comparison group and orange representing participants who experienced caregiving-related early adversity. (For interpretation of the references to colour in this figure legend, the reader is referred to the Web version of this article.)

Upon completion of Wave 1 of the MBB study, participants were invited to complete two follow-up sessions spaced approximately 12–18 months apart (Wave 3 is still in progress, see Fig. 1 Spaghetti plot for retention across the three waves of the study). Following the onset of the global COVID-19 pandemic (during Wave 1), data collection took place remotely over Zoom, while families were in their homes; prior to the pandemic, data collection occurred in the laboratory (*n* = 26; 15.7%).

#### 4.2.2 Attrition across waves

The achieved sample size in Wave 2 was *n* = 88, and the current sample size in Wave 3 is n = 72 (data collection for Wave 3 is ongoing). The attrition between Waves 1 and 2 (54%) was higher than anticipated, which we largely attribute to continuing issues related to the COVID-19 pandemic (e.g., have moved with no forwarding information, not interested in coming for in-person sessions, and difficulty balancing continuing shutdowns and work/school from home). The attrition between Waves 2 and 3 (<18% as Wave 3 data collection is ongoing) is in line with our expectations given the return to relative normalcy once COVID-19 was no longer classified as a public health emergency of global concern in May 2023.

#### 4.2.3 Impact of COVID-19 pandemic on data collection

**Fig. 2:**
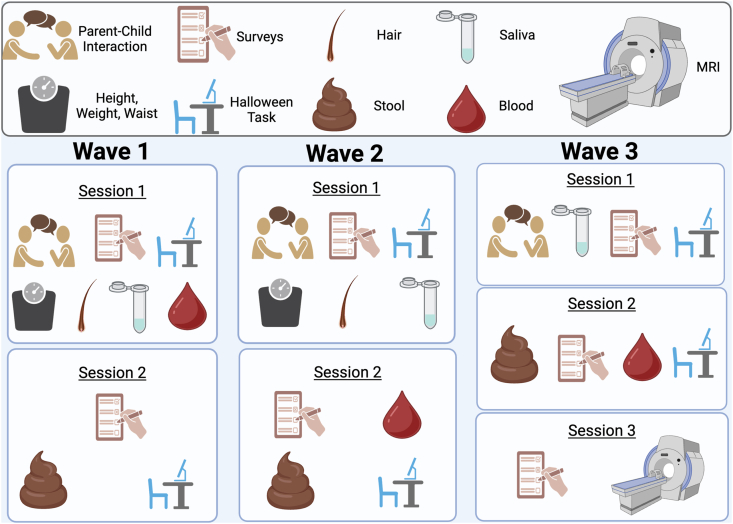
Tasks completed and measures collected in each study wave.

Enrollment for this study commenced in November of 2019, and n = 26 participants completed the study prior to the COVID-19 related research rampdowns at UCLA in May 2020. The study shifted to online data collection in April 2020, and the remaining participants completed the study online via Zoom with researcher guidance. Given the vast majority of the sample participated in the online format, we describe the procedure and measures used in the online format of the study in this protocol paper (see Fig. 2).

### 4.3 Questionnaires

Cronbach's alpha ratings for questionnaires ranged from acceptable (0.6–0.7), good and acceptable (0.7–0.8), good (0.8–0.9), and excellent (0.9–1.0) for all questionnaires (see Table 2 for more information)

**Table 2:** Cronbach's alpha for key study measures in the full sample (i.e., adversity-exposed and comparison groups). Alphas were acceptable (0.6–0.7), good and acceptable (0.7–0.8), good (0.8–0.9), and excellent (0.9–1.0). NA is indicated when measure was not administered at Wave 1.

Measure	Alpha	Score Mean (SD)	N
Security Scale (child self report)	0.80	3.1 (0.55)	162
Alexithymia (child self report)			
*Difficulty Identifying Feelings*	0.81	5.1 (3.7)	159
*Difficulty Describing Feelings*	0.71	5.0 (2.7)	158
*Externally Oriented Thinking*	0.41	6.9 (2.6)	158
CSSI (parent proxy report or child self report depending on child age)			
Parent Proxy Report	0.89	2.9 (3.7)	35
Child Self Report	0.90	13 (12)	125
Combined	0.91	10 (12)	160
BDI-II (parent self report)	0.90	0.35 (0.37)	131
CBCL(parent proxy report)^a^			
*Attention-Deficit/Hyperactivity (ADHD) Problems*	0.82	0.5 (0.58)	154
*Anxiety Problems*	0.83	0.28 (0.35)	154
*Conduct Problems*	0.84	0.11 (0.18)	154
*Depressive Problems*	0.80	0.2 (0.29)	152
*Oppositional Defiant Problems*	0.85	0.45 (0.48)	153
*Somatic Problems*	0.64	0.17 (0.24)	154
*Anxious/Depressed Problems*	0.85	0.27 (0.33)	154
*Withdrawn-Depressed Problems*	0.72	0.19 (0.27)	154
*Somatic Complaints*	0.72	0.16 (0.22)	153
*Social Problems*	0.80	0.25 (0.29)	154
*Thought Problems*	0.83	0.18 (0.25)	155
*Attention Problems*	0.88	0.18 (0.25)	154
*Rule-Breaking Behavior*	0.70	0.10 (0.15)	153
*Aggressive Behavior*	0.93	0.28 (0.33)	154
*Other Problems*	0.66	0.20 (0.20)	153
PedsQL - GI (parent proxy report)	0.96	0.44 (0.5)	154
PedsQL - Fatigue (parent proxy report)	0.92	1.00 (0.71)	154
PedsQL - Well-Being (parent proxy report)	0.90	3.43 (0.62)	154

a : shorter 25-item version of the RCADS self-report, which only generates a total score, was administered at Wave 3. For consistency across waves, the total score for the subset of items in the 25-item version was also calculated at Wave 2.

## 5 Childhood experiences

*Security Scale (Wave 1) – Child Self Report.* The Security Scale (Kerns et al., 1996) measures children's perceptions of security in their attachment relationship with their caregivers. The 15 items on the scale measure the degree to which children feel that they can rely on their caregivers in times of stress, their comfort and interest in communicating with their parents, and the degree to which they believe their parents are responsive and available for them. Scores on the measure range from 15 to 60, with higher scores indicating more perceived security in the relationship.

*Benevolent Childhood Experiences (BCEs; Wave 1) – Child Self Report.* The Benevolent Childhood Experiences (BCEs) Scale (Narayan et al., 2018) asked participants about the occurrence or presence of 10 positive experiences in their lives up to the age of 18 years. Items encompassed perceived relational and internal safety and security, positive and predictable quality of life, and interpersonal support. Total score of benevolent childhood experiences was computed by summing the number of all reported benevolent childhood experiences, thus higher scores indicated more benevolent childhood experiences.

*Traumatic Events Screening Inventory (TESI; Wave 1, 2, & 3) – Caregiver Proxy Report.* The Traumatic Events Screening Inventory - Parent Report Revised (TESI-PRR; Ghosh-Ippen et al., 2002) assesses a child's exposure to potentially traumatic events including those that are non-interpersonal (e.g., accident, natural disaster), interpersonal (e.g., abuse, neglect), and loss (e.g., death of a family member). Caregivers indicated whether their child had experienced each of the 24 stressors included in the instrument (Wave 2 and 3) over the time since last participation. In Wave 1, to reduce participant burden during COVID-19, we created a three-item version of the TESI, including just the items assessing physical abuse, sexual abuse, and neglect, and we asked about lifetime experience with those events. In all cases, higher scores indicate a child had experienced a higher number of traumatic events.

*Childhood Trauma Questionnaire: Short Form - Caregiver Self Report (CTQ; Wave 2).* The Childhood Trauma Questionnaire: Short Form (CTQ; Bernstein et al., 2003) was administered to caregivers to assess their own childhood experiences with six domains of potentially traumatic events, including physical, emotional, and sexual abuse, and physical and emotional neglect. Participants rated the intensity of each trauma experienced on a scale of 1 - “Never True” to 5 - “Very Often True”. We computed a total score on the CTQ by summing the intensities of all reported traumatic experiences to capture both the number of traumas experienced and their perceived impact; higher scores on the CTQ indicated higher trauma load.

## 6 Microbiome-related

*Bristol Stool Scale (Wave 1, 2, & 3) Caregiver Proxy Report:* Caregivers reported on the quality and consistency of the participant's stool sample from 1 (firm) to 7 (watery) using the Bristol Stool Scale (BSS) (O'Donnell et al., 1990). Briefly, caregivers and participants were provided with images and descriptions of different consistencies of stool and were asked to report the consistency of the sample on the day it was collected, as well as the typical consistency of the child's stool. This instrument also assessed whether the child was feeling ill on the day of collection, and whether the child's diet on the day of collection was typical of their regular diet, or significantly different from their regular diet.

*Microbiome Metadata (Wave 1, 2, & 3) Caregiver Proxy Report:* Caregivers reported on various potential microbiome confounders and moderators, such as the child's birth method, feeding method (i.e., breastmilk, formula, or a mixture) in infancy, perinatal antibiotic exposure, exposure to pets or livestock, country of birth, etc.

*Oral Health Questionnaire (Wave 2 & 3) Caregiver Proxy Report or Child Self Report:* Oral hygiene behaviors, oral health symptoms, history of access to dental care, and other potential oral microbiome covariates were assessed via an oral health questionnaire adapted from Simpson et al. (2020). The questionnaire was completed by caregivers, as well as by participants aged 9 or older.

## 7 Physical health

*Children's Somatic Symptoms Inventory (CSSI; Waves 1, 2 & 3) Caregiver Proxy or Child Self Report:* Somatic symptoms were reported using the CSSI (Walker et al., 2009). The CSSI was completed by caregivers of children younger than 8 years old, or by the participants themselves if they were aged 8 years old or older. The CSSI is a 24-item scale that asks how much in the past 2 weeks they were bothered by symptoms such as headaches, lower back pain, stomach pain, etc.

*Pediatric Quality of Life Inventory – Fatigue (Peds-QL-Fatigue; Waves 1 & 2) Caregiver Proxy and Child Self Report:* Caregivers (parent-proxy) and youth of all ages (self-report) completed the PedsQL-Fatigue (Varni et al., 2010) at Wave 1 and 2. The PedsQL-Fatigue measure contains three 6-item subscales that assess how often different dimensions of fatigue were a problem for the child in the past month: general fatigue, sleep/rest fatigue, and cognitive fatigue, on a scale from 0 (*never*) to 5 (*almost always*). All items were summed to create a total fatigue score, with higher scores representing greater fatigue. The measure demonstrated excellent reliability at Wave 1 (see Table 2).

*Pediatric Quality of Life Inventory – Gastrointestinal (Peds-QL-GI; Waves 1, 2, & 3) Caregiver Proxy and Child Self Report:* Caregivers (parent-proxy) and youth of all ages (self-report) completed the PedsQL-GI (Varni et al., 2015) at Waves 1, 2, and 3. The PedsQL-GI measure includes 10 individual scales assessing the following: stomach pain (6 items), stomach discomfort when eating (5 items), food and drink limits (6 items), trouble swallowing (3 items), heartburn and reflux scale (4 items), nausea and vomiting scale (4 items), gas and bloating scale (7 items), constipation (14 items), blood in poop (2 items), and diarrhea (7 items), on a scale from 0 (*never*) to 5 (*almost always*). All items were summed to create a total score, with higher scores representing greater gastrointestinal issues. The measure demonstrated excellent reliability at Wave 1 (see Table 2).

## 8 Caregiver stress

*Parenting Stress Index (Wave 1) Caregiver Self Report.* Caregivers reported on parenting-related stress using the Parenting Stress Index 4th Edition - Short Form (PSI-4-SF; Ríos et al., 2022), a 36-item instrument. Caregivers rated their agreement with a number of statements about their role as a parent (e.g., “*I feel trapped by parenting*”) on a five-point scale (e.g., 1 = “*Strongly disagree*” to 5 = “*Strongly agree*”). Higher scores indicate higher levels of parenting stress.

*Parental Stress Scale (Waves 1& 3) Caregiver Self Report.* Parenting-related stress was assessed using the Parental Stress Scale (PSS; Berry and Jones, 1995), an 18-item instrument that asks caregivers to rate their agreement with a number of statements related to parenting (e.g., “*Having children has been a financial burden*”) using a five-point scale (e.g., 1 = “*Strongly disagree*” to 5 = “*Strongly agree*”). Higher scores indicate a higher amount of parenting-related stress.

*Parenting Stress During COVID-19 (Wave 1) Caregiver Self Report.* An adapted version of the PSS at W1 was administered to assess parenting stress that was specific to COVID-19. Parents were asked to answer 9 items about parenting stress in the context of the COVID-19 lockdowns, using a scale of 1- “Strongly agree” to 5- “Strongly disagree”. Parents were also asked whether they agreed with a statement that they were more stressed as a parent because of the pandemic, and to endorse areas of their parenting that were causing them more stress because of the pandemic e.g., conflicts between child siblings.

## 9 Caregiver mental health

*Beck Depression Inventory (BDI-II; Waves 1, 2, & 3) Caregiver Self Report.* Caregivers reported on their depressive symptoms using the BDI-II (Beck et al., 1996), at each wave. Caregivers responded to 20 items using a 4-point scale from 0 to 3, and higher scores correspond to more severe depressive symptoms. One item regarding suicidality was omitted from the original scale for ethical reasons as study staff were not equipped to respond to endorsed suicidality. A mean of responses was obtained and used in primary analyses. Internal consistency (a conservative estimate of scale reliability) of the BDI-II was excellent at Wave 1.

*State-Trait Anxiety Inventory (STAI; Wave 2 & 3) Caregiver Self Report***.** Caregivers also completed a 10-item version of the STAI (Zsido et al., 2020) which assessed the severity of state (W2 & 3) and trait anxiety (W2) symptoms. Caregivers rated the severity of their symptoms on a four-point scale (e.g., 1 = “*Not at all*” to 4 = “*Very much so*”), and higher scores indicate more severe anxiety symptoms.

## 10 Youth mental health

*Child Behavior Checklist (CBCL; Waves 1, 2, & 3) Caregiver Proxy Report.* Caregivers reported on their child's mental health symptoms using the DSM-oriented depressive/affective, anxiety, attention-deficit/hyperactivity disorder (ADHD), oppositional defiant (ODD), conduct disorder (CD), and somatic problems subscales of the CBCL, version for children ages 6–18 years (Achenbach, 2001). A mean of responses for each DSM subscale was obtained and used in primary analyses. See *Supplement* for slight modifications made to the CBCL in our study including changes made to better reflect recent attitudes about the conceptualization of gender non-conformance.

*Revised Child Anxiety and Depressive Symptoms (RCADS; Waves 2 & 3) Caregiver Proxy and Child Self Report.* Caregivers and youth also reported on youth depressive and anxiety symptoms using the 47-item RCADS (Chorpita et al., 2000; Ebesutani et al., 2010, 2011) at Wave 2 and the 25-item RCADS (Ebesutani et al., 2017) at Wave 3. Caregivers and youth responded to the items using a 3-point scale from 0 to 2, and higher scores correspond to more frequent problems.

*KSADS Cross Cutting Symptom Questionnaire (KSADS-CCSQ; Wave 1) Caregiver Proxy Report.* The KSADS-CCSQ is a 23-item measure that is answered by parents for their children, assessing symptoms that cut across 12 psychiatric domains across the past 2 weeks: depression, anger, irritability, mania, anxiety, somatic, inattention, psychosis, sleep disturbance, repetitive thoughts and behaviors, and substance use. We omitted two items that assessed suicidal ideation and self-harm.

*Alexithymia (Waves 1 & 3) Child Self Report.* The alexithymia scale for children (Rieffe et al., 2006) was adapted from the adult Toronto Alexithymia Scale (TAS-20; Bagby et al., 1994). The 20 items on the scale measure the degree to which children experience alexithymia across three dimensions: difficulties identifying feelings, difficulty describing feelings, and externally oriented thinking. Children were instructed to score each item on a three-point scale (0 - “not true”, 1 - “a bit true”, 2 - “true”). Scores on this measure range from 0 to 40 with higher scores indicating higher levels of alexithymia. Internal consistency of the scale in this sample was good (See Table 2), with the exception of the externally oriented thinking subscale which has shown low internal consistency in various samples (see, Preece et al., 2018).

### 10.1 Biological and anthropometric samples/measurements

#### 10.1.1 Stool sample collection (wave 1, 2, & 3)

To index the gastrointestinal microbiome, stool samples were collected by participants in their own homes using Omnigene.gut stool collection kits (DNAGenotek) at each study wave, with their caregiver assisting as needed, and were then transported to the lab via mail. If a child reported recent short-term use of antibiotic, antifungal, or probiotic medication, or recent symptoms of acute digestive illness, sample collection was delayed until symptoms had resolved and/or medication had been stopped for at least two weeks. After collecting their sample, participants sent it back to the lab in a pre-paid mailer. The collection kits stabilize DNA at room temperature for 60 days and during postal mail transport (Evgueni Doukhanine et al., n.d.). Upon receipt of the sample in the lab, it was stored in a −80C freezer until DNA extraction and sequencing. Wave 2 and 3 stool samples have not yet been assayed.

#### 10.1.2 DNA extraction

The Bioinformatics Core at Arizona State University performed DNA extraction and sequencing of stool samples collected at Wave 1. Microbial DNA was extracted from samples using DNeasy PowerSoil Kit – (QIAGEN) following directions of the manufacturer. Illumina compatible Genomic DNA libraries were generated on an Eppendorf epMotion 5075 liquid handler using Kapa Biosystem's Hyper plus library preparation kit (KK8514). DNA was enzymatically sheared to approximately 300bp fragments, end repaired and A-tailed as described in the Kapa protocol. Illumina-compatible adapters with unique indexes (IDT #00989130v2) were ligated on each sample individually. The adapter ligated molecules were cleaned using Kapa pure beads (Kapa Biosciences, KK8002), and amplified with Kapa's HIFI enzyme (KK2502). Each library was then analyzed for fragment size on an Agilent's Tapestation, and quantified by qPCR (KAPA Library Quantification Kit, KK4835) on Thermo Fisher Scientific's Quantstudio 5 before multiplex pooling and sequencing using 2 lanes of an S4 300 flow cell on the NovaSeq platform (Illumina) at the Collaborative Sequencing Center (TGen).

#### 10.1.3 Bioinformatics

After sequencing, we followed Human Microbiome Project Consortium data processing guidelines (The Human Microbiome Consortium, 2012) to extract composition and functional potential information from the raw metagenome reads. Briefly, we first performed quality control using the KneadData v0.10.0 pipeline (Li et al., 2021). We then ran the remaining high-quality reads through the HUMAnN v3.7 pipeline (Beghini et al., 2021) for functional annotation of the metagenome and MetaPhlan v4.0.6 pipeline (Blanco-Míguez et al., 2023) for compositional analysis (see https://github.com/bablab/bablab_hoffman_metagenomics for scripts used in bioinformatics processing). Before downstream analysis, species and pathway abundances will be center log-ratio (CLR) transformed to bring them into unbounded space for statistical analyses (Quinn et al., 2019). Features with very low average CPM and/or low variation across samples were removed.

To meet the primary aims of the study, standard alpha and beta diversity metrics will be calculated. With respect to alpha diversity, we will assess Observed Taxa (Richness), Shannon Index, Simpson Index, Faith's Phylogenetic Diversity. Beta diversity will be assessed via weighted and unweighted UniFrac (phylogenetic distance), Jaccard Distance (prescence/absence similarity), and Bray-Curtis (abundance similarity). Given the rapidly changing nature of best-practices we will be using the most up-to-date recommendations for differential abundance analyses (Nearing et al., 2022).

#### 10.1.4 Blood samples (waves 1, 2, & 3)

To index inflammatory processes, dried blood spots were collected either in the lab by a trained researcher (see *Supplement*) or by participants in their own homes with live researcher instruction using Tasso M20 kits (TASSO-M20, n.d.). Participants gave samples up to three times. Due to small longitudinal sample and funding constraints blood was assayed once from each participant (i.e., participants who provided blood at Wave 1, did not have blood assayed at Wave 2 or 3). After collection and receipt by the lab, samples were stored in a −80C freezer until assayed. If a child reported a recent infectious illness or temporary use of inflammation-altering medication, sample collection was delayed until at least 2 weeks after symptoms resolved, or medication was stopped.

The Tasso M20 kit is a self-contained device designed for capillary blood sample self-collection in a home setting. Each participating caregiver was instructed to stick the kit's adhesive side to their child's arm and then press a button which triggered a retractable lance to break the skin, drawing four 20-μl drops of blood onto an absorbent card that was stored inside an attached card holder (Solheim et al., 2021). Once the card was full or after 5 minutes had elapsed (whichever came sooner), the caregiver was then instructed to peel the device from the child's arm, breaking the bottom card holder away from the seal and lance. The participant was then instructed to seal the card in a foil bag and store it at room temperature, before shipping back to the lab within 24 hours of collection. No separate drying step is required for Tasso M20 kits.

*Processing of Inflammatory Assays*. Blood samples were sent to the UCLA Social Genomics Core to assess genome-wide transcriptional profiles using RNAseq (i.e., expression of immune-related genes) (Cole, 2019). After RNA extraction, samples were shipped to Lexogen for sequencing, which includes conversion to cDNA libraries using the QuantSeq3′ FWD mRNA-Seq LibraryPrepKit for Illumina (Lexogen Inc) and sequencing using an Illumina HiSeq4000 instrument (Illumina Inc), following the manufacturers' standard protocol for low mass RNA samples. Sequencing data will be evaluated for endpoint quality assurance thresholds for dried blood spot RNAseq samples (>10 million single-strand 65-nucleotide reads per sample, >90 % of reads aligning to the reference human genome, correlogram average profile ≥0.80).

Planned analyses will examine inflammatory gene expression using an a priori specified set of 19 pro-inflammatory gene transcripts (e.g., *IL1β, IL6, IL8/CXCL8, COX2/PTGS2, TNF*) that have been previously linked with early life caregiving adversity (Marie-Mitchell and Cole, 2022)—the Conserved Transcriptional Response to Adversity (CTRA). The total number of reads for each gene will be normalized to transcripts per million (TPM) total mapped reads, floored at 1 TPM, and log2 transformed for analyses. Reads with insufficient sample variance in expression (i.e., standard deviation of expression <0.5 units in log2 metric) will be removed. The remaining reads will be standardized within-gene and averaged to create a composite score of pro-inflammatory gene expression.

#### 10.1.5 Hair samples (wave 1 & 2)

To index cortisol, hair samples were collected (collection and processing methods were previously described (Gancz et al., 2024). Briefly, under guidance from a trained researcher, caregivers collected the child/adolescent participants' hair from underneath the crown of the head, cut near the root. Most of the Wave 1 samples (n_crEAs_ = 68, n_comparison_ = 79) and all of the Wave 2 samples were stored at room temperature, but N = 4 samples were stored at −20C and thawed before processing at Wave 1. For Wave 2, a total of 77 hair samples were collected (crEA group n = 29 and Comparison group n = 48). Wave 1 samples were shipped to the Meyer lab (Arizona State University) at ambient temperature where they were then processed and analyzed according to the methods described in Meyer et al. (2022) with minor modifications, see *Supplement* for more. Wave 2 hair samples have not yet been assayed.

#### 10.1.6 Saliva samples (wave 1, 2, & 3)

To index the oral microbiome, saliva samples were collected (collection and processing methods were previously described (Gancz et al., 2024). In brief, participants refrained from ingesting food or water for 30 minutes, then collected saliva samples with help from caregivers and guidance from a trained researcher using OMNIgene® ORAL sample collection and stabilization kits, per manufacturer protocols. In total, 152 participants (crEA n = 66, n = 86 Comparison) provided saliva in Wave 1. One sample was collected via a swab and the rest were collected via passive drool. Five samples (n = 3 crEA, n = 2 Comparison) were frozen at −20C directly in the collection tube. Remaining samples were incubated at 50C for 2 hours, vortexed, and frozen at −80C in cryovials. See *Supplement* for information with respect to sample processing.

#### 10.1.7 Height/weight/waist circumference (wave 1, 2, & 3)

Measurements of children's height, weight, and waist circumference were obtained in Waves 1 and 2. Families were provided with a measuring tape and were instructed on how to use it to measure children's height and waist circumference during their study session. Current weight was determined using a scale if the family had one in their home; otherwise, a recent estimate (e.g., from a physician visit) was used. Body mass index (BMI) was calculated from height and weight to be used as a covariate, as it is associated with gut microbiome development (Murugesan et al., 2018), inflammation (Slopen et al., 2013), and the relations between early life adversity, inflammation, and internalizing symptoms (Chiang et al., 2017, 2022). In Wave 3, participants were weighed on digital scales before entering the scanner.

### 10.2 Tasks

#### 10.2.1 Halloween memory task (waves 1, 2, & 3)

**Fig. 3:**
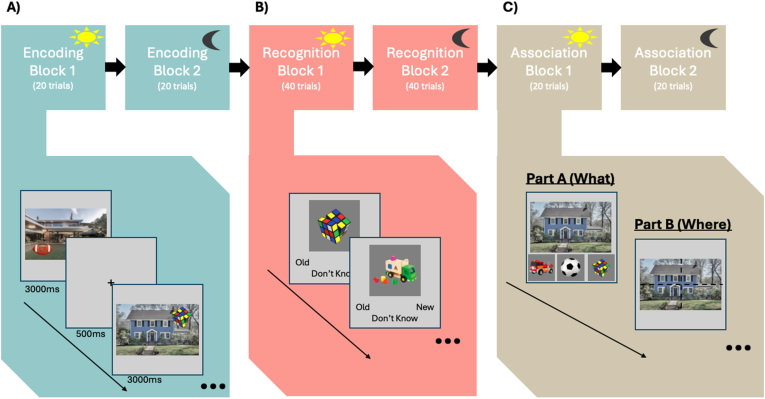
Halloween memory task design. A) Encoding blocks B) recognition blocks C) association blocks.

Participants completed the Halloween Memory Task (see Fig. 3). The task was designed to assess the influence of affectively valanced contexts on spatial associative memory. The encoding phase involved presenting participants with series of either affectively neutral or negative contexts (houses and outdoor scenes), before items (candy and toys) appeared superimposed over the background image, in one of four quadrants (bottom left or right, top left or right). The participants were told that they were trick-or-treating and they had to remember where they saw each toy or candy. The background contexts were presented in one of two blocks (Day Block – affectively neutral, and Night Block – affectively negative), which were counterbalanced (see Fig. 3A). The affectively neutral background contexts included photographs of house interiors, front and back yards taken in the daylight, and found via internet searches. The affectively negative background contexts included photographs of building and house interiors and yards, and woods, which were spooky or haunted looking, e.g., included spiderwebs, represented nighttime, and were dark. These images were also found using internet searches.

Within each of the Day and Night blocks, the order and timing of stimulus presentation was the same: First, a background context was displayed on the screen. After 500ms an item (toy or candy) was superimposed over the background context in one quadrant, where it then stayed on the screen for an additional 2500ms, before both the item and background context were replaced with a fixation cross, which signaled the end of the trial (total trial time was thus 3000ms). Each block consisted of 20 unique trials. Of those trials, 10 featured a toy and the other 10 featured a candy superimposed over the background contexts. Each background context was paired with one toy/candy item. Each context-item pair was presented once during the encoding phase, with no background being repeated, resulting in a total of 40 unique backgrounds paired with 40 unique items across both blocks.

After encoding, participants took part in two self-paced memory tests (Fig. 3B). The first of these tests took place immediately after encoding, and the second took place approximately two weeks after encoding. In each of these tests, there were two components: a recognition memory test, and associative memory test. In the recognition test, participants were presented a series of items (toys or candy) from the encoding phase (targets) mixed with items from the same categories that were not presented in the encoding phase (foils). Participants were asked to identify whether they had seen each item before by selecting ‘old’ if they had seen the item before, or ‘new’ if they had not. Participants were discouraged from guessing and instead encouraged to choose ‘don't know’ when unsure. After making their selection, participants were then shown a screen asking how confident (“sure”) they were in their selection on a 4-point scale, from 1 = “not sure”, to 4 = “very sure”. The presentation of the items during the recognition test were also blocked and presented in the same order as encoding. That is, if the participant saw the Day block first during training, then the Day test was presented first (Day targets intermixed with foils), followed by the Night test (Night targets intermixed with foils). Each of the two blocks consisted of 40 trials (20 targets and 20 foils).

For the association test, each trial had two parts (A and B; Fig. 3C). In part A, participants viewed a background context seen during encoding with three target items (all from the same category, either toys or candy) presented underneath the context. One of the three items had been paired with that background context during encoding (i.e., was correct). Participants were asked what item had been paired with the background context during the encoding phase. Participants selected the item that had been paired with the context during encoding. Once they selected an item, part B of the trial was initiated. In part B, a grid was overlaid on the house, dividing it into four quadrants. Participants then indicated in which quadrant they had previously seen the selected item. This allowed for memory performance to be assessed at coarse (correct item-context pairing) and detailed (correct-item-context-location grouping) level of analysis. The associative memory task was comprised of two blocks of 20 trials each, with the order of presentation matching the encoding and recognition phases.

#### 10.2.2 Spatial memory task with reward component (wave 3)

**Fig. 4:**
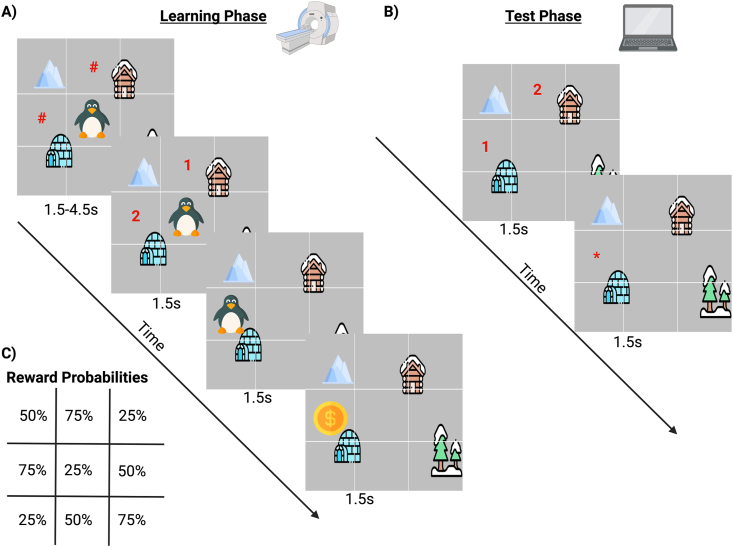
Map Reward Task Design. A) Participants completed the learning phase of the Map Reward Task inside the scanner. They were presented with two options of where to move the penguin. After moving the penguin, participants saw whether or not they received a reward. Participants completed two runs (36 trials each) of the learning phase. B) After the MRI scan, participants completed the test phase of the Map Reward Learning Task. They were presented with two options and had to decide which of the two had more rewards during the learning phase. A “∗” was presented to confirm their choice, but no feedback on the correctness of their choice was provided. C) A table of the reward probabilities of each part of the map, which was constant for all participants.

Participants completed the Map Reward Memory Task as part of the in-person MRI session. The task was adapted from an existing spatial reward learning paradigm (Calabro et al., 2023; Parr et al., 2021). The learning phase was completed inside of the scanner, and the memory test phase was completed outside of the scanner (Fig. 4).

During the learning phase, participants were instructed that they would be exploring a 3x3 grid map with a penguin in order to find rewards (Fig. 4A). First, participants were shown two “#”s in different squares on the grid as options of where to move on the map (1.5s–4.5s). The “#”s were then replaced with a “1” or “2” and participants could use the button box keys (index finder for 1, middle for 2, on a 4-botton box) to select where to move (1.5s). The penguin was then moved and displayed at the new location (1.5s). Then the trial outcome was displayed (1.5s) showing either no reward (a blank square), a small reward (one gold coin; 75% of rewards), or a big reward (multiple gold coins; 25% of rewards). All locations on the map had a fixed chance of having a reward (25%, 50%, or 75%; Fig. 4C) and the choices presented always had unequal probabilities of reward (e.g., 25% vs. 75% chance). The participants completed two runs of the learning phase in the scanner (36 trials each) during which the chance of receiving a reward at each location of the map remained constant.

The *test phase* of the task was completed outside of the MRI scanner (Fig. 4B). Participants were instructed to choose which of two map locations were associated with a greater probability of rewards in a series of forced choice trials. For each trial, the participants were shown two locations (indicated with a “1” and a “2”) and were asked to select the location with the bigger reward (this time using the “1” and “2” keys on the computer). Their answer choice was confirmed by a “∗” on the location they selected (1.5s). The *test phase* consisted of one run of 27 trials, and no feedback on their performance was provided.

#### 10.2.3 Caregiver-child interaction (waves 1, 2, & 3)

Youth and their caregivers completed videorecorded caregiver-child interactions, which included a conflict resolution task followed by a pleasant event-planning task. In the conflict resolution task, the participants were given 1 minute to select area(s) of conflict from the Issues Checklist (Prinz et al., 1979) and were then instructed to spend 5 minutes discussing those source(s) of conflict and to generate solutions. In the subsequent pleasant event-planning task, the participants were given 1 minute to choose a pleasant activity or activities from the Pleasant Events Checklist (MacPhillamy and Lewinsohn, 1982) and were then instructed to spend 5 minutes trying to plan the pleasant event(s). The order of tasks was fixed as we conceptualized the pleasant event-planning task as a recovery from conflict period, with the intended aim of reducing overflow of negative emotions potentially generated during the conflict resolution task to the rest of the data collection session.

The videorecorded session from the Caregiver-Child Interaction, was behaviorally coded using the Family Interaction Macro-coding system (FIMS; Holmbeck et al., 2002; Richmond et al., 2020). The FIMS coding manual was used to rate caregiver and child affect and behavior during each of the two interaction tasks (conflict resolution task, pleasant event-planning task). The FIMS is a global coding system, with 31 individual items that represent positivity/warmth (e.g., verbal warmth, supportiveness), social communication (e.g., requesting input from other family members, promoting dialogue and collaboration), and negative behaviors (e.g., withdrawal from conflict, pressuring others to agree), with each item coded separately for the child and their parent (Richmond et al., 2020). For each item, behavior is rated on a 5-point Likert-type scale (1 = not at all; 2 = rarely; 3 = sometimes; 4 = frequently; 5 = very often). Prior to coding the videorecorded interactions, coders participated in extensive training (see Supplement for details and coder reliability analyses).

### 10.3 Neuroimaging sequences (Wave 3)

#### 10.3.1 Overview

All neuroimaging data were collected during Wave 3 of the study. Images were acquired using a 3T Siemens Prisma Scanner with a 32-channel head coil. All neuroimaging sessions took place at the UCLA Brain Mapping Center.

#### 10.3.2 T1 anatomical sequence

The high-resolution T1-weighted scan was optimized for gray-white contrast. The following parameters were used: 2300ms repetition time (TR); 2.26ms echo time (TE); 8° flip angle (FA); 256 x 256 in-plane resolution; 192 sagittal slices (1.0mm thickness).

##### 10.3.2.1 Functional (f)MRI sequences

All functional images were acquired on the transverse plane using an echo-planar sequence with the following parameters: 1500 ms repetition time (TR); 30.0 ms echo time (TE); 70° flip angle (FA); 192mm × 192mm field of view (FOV); 36 slices; 3.0mm slice thickness; 2.0 x 2.0 × 3.0 mm voxel size). For the resting state fMRI scan, 273 vol were acquired (Acquisition Time = 7:00 minutes), during which participants viewed the Inscapes movie (Vanderwal et al., 2015). For the Map Reward Learning Task, 173 vol were acquired for each run of the task (Acquisition Time = 4:30 minutes, per run).

##### 10.3.2.2 Neuromelanin sequence

The T1-weighted Fast Spin Echo (FSE) scan was collected to estimate neuromelanin using the following parameters: 750 ms TR; 12.0 ms TE; 120° FA; 220 mm × 220 mm field of view (FOV); 11 slices; 2.5 mm slice thickness; 0.4 x 0.4 × 2.5 mm voxel size; Acquisition Time = 3:44 minutes). Prior to the start of the scan the FOV was manually aligned by trained operators to be centered on the locus coeruleus (LC), a key region associated with attention and stress responses that interacts with the hippocampus and amygdala (Tanaka et al., 2000). This sequence assess levels of neuromelanin, a proxy measure of dopamine, in the LC (Wakamatsu et al., 2015).

## 11 Discussion

Extensive research has demonstrated that early life adversity (ELA) in general, and caregiving-related early adversity (crEA) in particular, are associated with increased risk for mental and physical health problems throughout the lifespan. Much of the research attempting to understand the mechanisms underlying such increased risk has focused on behavioral pathways as well as the central nervous system (Gee et al., 2014; Nachmias et al., 1996; Seltzer et al., 2012). Less appreciated is the impact of crEA on the development of peripheral health systems, which may interact with the brain and behavior, to further define risk and resilience. One pathway by which central and peripheral systems interact, and which has received increasing attention in recent years, is the brain-gut-microbiome axis. While this axis has been consistently linked to a variety of mental and physical health outcomes, limited data on how crEA affects the functioning of this pathway exists. The aim of the Mind, Brain, and Body (MBB) study is to elucidate those connections to derive a deeper understanding of the many factors that impinge upon development to influence trajectories towards health or disorder. Building upon the literature linking crEA to altered memory and hippocampal development, reward responsivity, and immune health (Callaghan et al., 2019; Dillon et al., 2009; Hantsoo et al., 2019; VanTieghem et al., 2021), the MBB study is the first to assess the role of microbiome development in those processes, and to explore how these nodes of the brain-gut-microbiome axis, cognitive, affective and immune development, interact to modulate vulnerability for internalizing psychopathology in at-risk populations of youth.

While ELA exposure is a general risk factor for psychopathology, evidence strongly supports a role for crEA specifically in shaping development of neural regions important in our affective world – the amygdala, hippocampus, and striatum (Vannucci et al., 2023). The proposed importance of caregiving-related adversities for shaping affective development stems from the fact that caregivers (who function most prominently in children's early social relationships) form the context in which infant brains learn to conceptualize information about the body and environment and use it in a way that guides predictive self-regulation – allostasis (Atzil and Barrett, 2017; Sterling, 2012). In other words, development within social relationships is an evolutionarily preserved feature of human life which makes early relationships especially salient for shaping brain and peripheral system development (Atzil et al., 2018). Indeed, extensive evidence has demonstrated that parents are critical in supporting affective regulation throughout childhood, and that disruptions to caregiving exert powerful developmental effects on the child that can last throughout the lifespan (Callaghan & Tottenham, 2016a, 2016b). Given these data, the MBB study focuses on crEA, as an especially salient input to children's affective development.

Through the use of a longitudinal design, and concurrent measurements of microbiome, immune, behavior, and the brain, the MBB study will answer several important questions which may ultimately be used to guide prevention and treatment for crEA-associated impacts on children's development. Specifically, results will provide more detailed information on the structure and function of the microbiome in middle childhood and adolescence, and how it changes across those stages of life, which remain extremely understudied developmental epochs in the microbiome literature to date. Moreover, the results will provide crucial insights into how the gut-microbiome is associated with developmental changes in memory and reward processes as well as hippocampal and striatal function, both of which have been extensively implicated in the progression and maintenance of internalizing disorders (Aikins and Craske, 2001; Dahmen et al., 2017), and linked to the microbiome in animal models (Dandekar et al., 2022; De Santa et al., 2024; Peng et al., 2019; Zhu et al., 2022). Finally, the data will enable assessment of how caregiving-related adversity impacts immune functioning through gut microbiome pathways. Given the prevalence of internalizing disorders in youth have increased by 29% from 2016 to 2020 (Lebrun-Harris et al., 2022), and exposure to early life stress and caregiving-related adversity is common (NCHS, 2024), improving our understanding on how early adversity alters various cognitive and biological systems, and how those systems interact to increase risk, is imperative for better understanding the development and disease course of internalizing psychopathology.

An overarching goal of our work is to elucidate the pathways by which the gut microbiome can be targeted in prevention and intervention for mental illness, especially after adversity exposure. Notably the gut microbiome undergoes a sensitive period of development in early childhood, that is concurrent with, and sometimes precedes, sensitive developmental periods for several neural circuits, immune systems, and memory processes (Aburto and Cryan, 2024; Callaghan, 2020). Thus, identifying when and how the early microbiome may influence later developing neural and cognitive networks is essential for determining its utility as a target for internalizing psychopathology. As the gut microbiome can be easily manipulated (via diet, prebiotics, probiotics, etc.) it serves as a non-invasive and potentially cost-effective adjuvant to boost the effectiveness of current first-line treatments for mental illness. Despite the promise of the microbiome in the therapeutic space, much remains to be understood about the mechanisms by which it effects various aspects of physical and mental health, which limit its utility and the specificity with which it can (or should) be manipulated. Our hope with the Mind, Brain, and Body study is to grow the mechanistic body of literature examining how the gut microbiome contributes to the development of internalizing psychopathology, especially in at-risk youth.

## Funding

This work was supported by the 10.13039/100000025National Institutes of Mental Health (NIMH) of the 10.13039/100000002National Institutes of Health [R00MH113821, B.L.C and T32MH01575, F.R.Q]; the UCLA Faculty Career Development Award (B.L.C.); and the Microbiome Center Seed Fellowship (F.R.Q. and N.G.)

The content is solely the responsibility of the authors and does not necessarily represent the official views of the National Institutes of Health.

## CRediT authorship contribution statement

**Shiba M. Esfand:** Writing – review & editing, Writing – original draft, Visualization. **Francesca R. Querdasi:** Writing – review & editing, Writing – original draft, Visualization, Data curation. **Naomi N. Gancz:** Writing – review & editing, Writing – original draft, Visualization, Data curation. **Paul W. Savoca:** Writing – review & editing, Writing – original draft, Visualization. **Siyan Nussbaum:** Writing – review & editing, Writing – original draft, Visualization, Project administration, Data curation. **Jennifer A. Somers:** Writing – review & editing, Writing – original draft. **Julia Ditzer:** Writing – review & editing, Writing – original draft. **Matthew B. Figueroa:** Writing – review & editing, Writing – original draft. **Kristen Chu:** Project administration, Data curation. **Emily Towner:** Project administration, Data curation. **Bridget L. Callaghan:** Writing – review & editing, Writing – original draft, Supervision, Methodology, Funding acquisition, Conceptualization.

## Declaration of Competing interest

All authors have no conflicts of interest or relevant disclosures.

## Data Availability

Data will be made available on request.
