# Supplementary material for: The mind, brain, and body study: A protocol for examining the effects of the gut-brain-immune axis on internalizing symptoms in youth exposed to caregiving-related early adversity
Source: Brain Behav Immun Health. 2024 Oct 5;42:100880. doi: 10.1016/j.bbih.2024.100880 (PMC11776082; doi:10.1016/j.bbih.2024.100880)
Supplement: Multimedia component 1 [file mmc1.docx]

**Supplement**

***Inclusion/Exclusion Criteria for crEA and Comparison Group***

Parents were eligible to participate in the Mind, Brain, Body study if they were aged 18 years or older, had at least one child between 6-16 years of age, and could read and write in English. Youth were eligible to participate if they were fluent in the English language; exclusion criteria for youth included uncorrected vision and caregiver-report of youth behavioral or psychological concern, learning disability, or youth-reported illicit substance use that would interfere with their ability to comply with study procedures. The UCLA IRB approved all study procedures prior to study inception.

Half of children in the sample had continuously lived with their biological/birth/first parents and had never experienced maltreatment (Comparison group; *n*=90; 55%); the other half of children had experienced significant early life caregiving-related adversity in the form of removal/surrender from their biological/birth/first parents’ care and/or maltreatment by a caregiver (Caregiving-related Early Adversity [crEA] group (*N*=73; 45%). Within the crEA group, some children were either adopted at birth or placed in foster care before they were adopted later in development (*n* = 41), others experienced placement in foster care before entering a Guardianship care arrangement (which typically results in adoption) (*n* = 17), another was maltreated by a caregiver not resulting in foster care (*n*=1), and some were exposed to institutional/foster care abroad before being adopted by US families (*n =* 14).

***Observed Caregiver and Child Behavior – Coder Training and Reliability***

Following an initial tutorial with an experienced coder, coders independently coded five interactions from an independent training dataset for reliability assessment and achieved a minimum 90% agreement rate (i.e., no more than a one-point difference) on practice videos before coding for the present study. Teams of two trained coders double-coded each videorecorded task for caregiver and child behavior. Intraclass Correlation Coefficients (ICCs) were checked upon completion of coding of all interactions, reflecting absolute agreement in scores when subjects were rated by multiple coders chosen at random from a larger population of possible coders and the average of their ratings was used (Shrout & Fleiss, 1979). On average, interrater reliability was moderate for parents (ICCconflict = 0.57, ICCpositive = 0.54) and children (ICCconflict = 0.67, ICCpositive = 0.65) during the conflict resolution task and pleasant event-planning tasks. Final item scores were obtained by averaging the two coders’ scores.

***Saliva Sample Processing (Wave 1)***

Wave 1 samples were shipped to the sequencing site (Arizona State University lab services) on dry ice. The V4 region of the 16S gene was by Arizona State University lab services sequenced using the 515f/806r primer set [(Caporaso et al., 2011)](https://www.zotero.org/google-docs/?MOLAfH) following the Earth Microbiome Project (EMP protocol). PCR amplifications for each sample were conducted in duplicate, then pooled and quantified using an accublue kit. To control for extraneous contamination, library preparation included a no template control. 240ng of DNA per sample were pooled and cleaned using QIA quick PCR purification kit (QIAGEN). Then, the pool was quantified using the qubit. The DNA pool was subsequently diluted to 4 nM, then denatured and diluted to 4 pM with a 25% of PhiX. The DNA library was then loaded in the MiSeq Illumina and run using the version 2 module, 2x250 paired-end, following the manufacturer directions. Three samples from the crEA group were not successfully sequenced during the first run but were subsequently re-run successfully. Wave 2 and 3 saliva samples are all collected using passive drool and incubated at 50C for 2 hours, vortexed, and frozen at -20C in cryovials. They will be sequenced using shotgun metagenomic sequencing at the UCLA Microbiome Core facility.

***Hair Sample Processing***

Each hair sample was weighed, washed with isopropanol twice to remove contaminants, then air-dried. Washed samples were ground to a fine powder using a bead mill, extracted overnight into methanol, and centrifuged to spin down the beads and the powdered hair. An aliquot of the methanol extract was transferred to a clean tube, dried using a vacuum evaporator, and then reconstituted in assay buffer.

Reconstituted extracts were spin-filtered to remove any residual particulate material, then assayed in duplicate along with standards and quality controls using the Arbor Assays DetectX Cortisol ELISA kit. Intra- and inter-assay coefficients of variation for this assay were both <10%. In total for Wave 1, 143 hair samples were collected (64 from the CA group and 79 from the Comparison group), of which all but 1 (from the Comparison group, which was too small to process) were assayed for cortisol levels.

***In Person Blood Sample Collection (Wave 1)***

In-person dried blood spot collection was performed by a trained researcher who massaged the middle or ring finger on the participant’s non-dominant hand, and then used a BD Microtainer contact-activated lancet to prick the selected finger. Five drops of blood were then placed onto each of two Whatman 903 Proteinsaver cards (GE Healthcare Bio-Sciences). Blood spot cards were then dried overnight, sealed, labeled with ID number and date, and placed in a small plastic bag with a desiccant pack before being frozen.

***Gut Microbiome Sequencing***

Microbial DNA was extracted from samples using DNeasy PowerSoil Kit – (QIAGEN) following directions of the manufacturer. Illumina compatible Genomic DNA libraries were generated on an Eppendorf epMotion 5075 liquid handler using Kapa Biosystem’s Hyper plus library preparation kit (KK8514). DNA was enzymatically sheared to approximately 300bp fragments, end repaired and A-tailed as described in the Kapa protocol. Illumina-compatible adapters with unique indexes (IDT #00989130v2) were ligated on each sample individually. The adapter ligated molecules were cleaned using Kapa pure beads (Kapa Biosciences, KK8002), and amplified with Kapa’s HIFI enzyme (KK2502). Each library was then analyzed for fragment size on an Agilent’s Tapestation, and quantified by qPCR (KAPA Library Quantification Kit, KK4835) on Thermo Fisher Scientific’s Quantstudio 5 before multiplex pooling and sequencing using 2 lanes of an S4 300 flow cell on the NovaSeq platform (Illumina) at the Collaborative Sequencing Center (TGen).

***Questionnaires continued***

**Questionnaire Scoring and Attention Checks**

Questionnaires were scored according to scoring manual instructions specific to each measure, using R in RStudio. Attention checks were included throughout the study questionnaires (e.g., “Please select ‘A little’ if you are paying attention). There were 5 attention checks in Wave 1, 10 attention checks in Wave 2, and 4 attention checks in Wave 3. In Wave 1, participants passed 97.50% of attention checks on average. In Wave 2, participants passed 97.24% of attention checks on average.

**CBCL Modifications**

Caregivers responded to the items comprising the depressive/affective, anxiety, attention-deficit/hyperactivity disorder (ADHD), oppositional defiant (ODD), conduct disorder (CD), and somatic problems subscales using a 3-point scale from 0 to 2, and higher scores correspond to more frequent problems. In the present study, two items regarding suicidality were omitted from the affective/depressive scale for ethical reasons. Additionally questions regarding gender non-conformance were altered to better approximate gender dysphoria. Specifically ‘Behaves like opposite sex’ and ‘Wishes to be of the opposite sex’ included the following language added on ‘AND such behavior causes child significant distress or impairment in social, school, or other important areas of functioning.’ At Wave 1, four items were accidentally omitted, including from the depressive/affective (little interest in activities), ADHD (fails to finish, inattentive), and CD (breaks rule) assessment batteries. Caregivers’ responses on the CBCL were also used to derive broadband internalizing and externalizing subscales. Internal consistency of each scale was acceptable or better, at each wave, except for the somatic symptoms subscale at Wave 2.

***Interoception***

**Multidimensional Assessment of Interoceptive Awareness - parent (W3).** To assess interoceptive sensibility in the parent, we used the 37-item Multidimensional Assessment of Interoceptive Awareness (MAIA-2), which examines eight distinct dimensions of interoceptive sensibility: Noticing, Not-distracting, Not-worrying, Attention Regulation, Emotional Awareness, Self-regulation, Body Listening, and Trusting [(Mehling et al., 2018)](https://www.zotero.org/google-docs/?BjfavX). There is no interpretable combined total score on this measure.

**Multidimensional Assessment of Interoceptive Awareness - youth (W3).** Interoceptive sensibility of the child was assessed with the 32-item MAIA Youth version (MAIA-Y; [(Jones et al., 2021)](https://www.zotero.org/google-docs/?hXkHSx). The MAIA-Y has modified language to be more appropriate for use in youth populations and has been previously validated in a sample of 7-17 year olds. The MAIA-Y generates subscale scores for the same eight dimensions of interoceptive sensibility that the *MAIA-2* generates.
